# Drs. Watt and Taft on the First Permanent Molars

**Published:** 1883-01

**Authors:** 


AMERICAN JOURNAL

OF

DENTAL SCIENCE.

Vol. XVI. THIRD SERIES—JANUARY, 1883. No. 9.

### ARTICLE I.

### *Drs. Watt and Taft on the First Permanent Molars.*

[Proceedings of the Ohio State Dental Society.]

Dr. Watt; The question comes up with every young mother, and I guess every old mother, what shall be done with the baby; and in general we just simply say, nurse it. The baby ought to be taken care of, and I don't know that we can say anything better in regard to the six year molars. Just how the baby is to be nursed, whether to be fed on goat's milk, or cow's milk, or tug at its mother and make its honest natural living, depends on the circumstances, whether the mother can support it that way or not—whether her mammary glands secrete a health-giving fluid—whether the state of her constitution will tolerate this. Whatever, though, is to be best for the baby and all others concerned, is to be done. So these six year molars ought to be taken care ot. There is not much doubt that our Heavenly Father intended that they should stay there until the owner of them shall die of old age. I think you may dissect the human body from end to end, you may make a microscopic investigation, and you can't find any preparations for death. Everything in a healthy constitution—every organ, if it is healthy—points to life, and not to death.

And the first permanent molars—the six year molars, as they are sometimes called—are no exception to the rule. They are made to last until natural death ensues from simply the running down of the clock; and, therefore, they are to be treated accordingly,—that is, provided their constitution is normal. Sometimes, however, in one sense there is no physiology at all, but all is pathology, for man is a fallen being; theretore, the first permanent molars often suffer. It is a great misfortune that the child is often shut up in confined rooms, and an unhealthy atmosphere, at the time these teeth are being developed, and they come with a feeble, defective structure, and hence they decay readily and easily. Another trouble, however, is that about the time these teeth are fairly through the gum, and before they have had time to consolidate, the child is sent into school, and there is kept for about eight hours a day, and must not move—it must be in dread; and it is not one time in ten that you can pass through a primary school and, with the eyes of a physician, see one healthy child in the school room of thirty to fifty children. It is just as much as a bargain if the public schools, as now conducted, escape being a nuisance. I am inclined rather to think that they are a nuisance, with all the glory that we have attached to them, and our talk about "free education." Children are forced into unnatural conditions. Now, that is not a good way to treat these first molars. When I get to be the autocrat of all the Americas, a child that is under eight years shall not be shut up in school at all, and it shall not be shut up more than a half hour to an hour at a time, varying with the constitution, if eight; and the teacher that assigns lessons that have to be studied at home, and then simply recited in the school room—to be studied at home, when the child ought to feel as free as a colt turned out on pasture, or as a lamb by the still waters—shall be summarily dealt with. The child is in dread, is weighed down with the consciousness that here is a series of long lessons. The child of eight years old is usually taxed further, in regard to brainwork, than the student of twenty-five in the university. This is not doing the first molars any good. The result is that the secretions become depraved, even if the little child, with an ammoniacal degeneration of tissue. If you desire a friendly kiss from even a small child, you will find the breath loaded with ammoniacal putrefaction. It does not smell much like spirits of hartshorn; hence, people don't think it is ammonia. They forget that the ammonia is combined, under such circumstances, with phosphuretted hydrogen. The brain and nervous system of the child are actually burned out. Hence, we find a far more offensive smell than ammonia would itself give. And we find that condition in the child because it is deprived of fresh air; it is loaded with anxious care; it has no hours that it feels free from the heavy responsibility, and no time at which it is allowed to feel like a child. The consequence is we find all the secretions depraved. When the mouth is in this condition, these teeth, being soft, corrode. And parents, inasmuch as these teeth have made their appearance before the incisors are shed, think they are temporary teeth. And if there is one thing that parents must be far more generally taught than another, it is that these are permanent teeth, and must be looked after. The secretions of the mouth must be kept healthy. And, in order to do this, the child must be treated as a rational human being.

But there are exceptions to all of this. Sometimes, on account of our race being wonderfully mixed, the child may inherit its jaws from one race and its teeth from another. The teeth are secretions. The secretory organs are more likely to be inherited from the mother than the father. The anatomical structure of the head and the spinal cord are more liable to be inherited from the father. You can always remember this if you look at the mule; its head and back are almost like its father's, while its size and the secretory organs are like those of the mother. So the child, which is more likely to have the head of the father, often takes the teeth from the mother. If the mother happens to be of a race or nationality that usually has large teeth, and the father happens to be one of the finer featured races, the result will be greater disproportion. Now, then, if these teeth are found decayed, if there is not room for all the teeth, and those teeth are so depraved that they can not be retained permanently, then remove all four of them ; especially if the patient is a little girl, and you don't want her beauty marred, have them all taken out at one time. Sometimes, by removing these, there is room for the teeth to place themselves in their proper arch, and when they have room thus to make their appearance, they are to be preserved by appropriate filling in time and the proper protective treatment, which means the general health of the child.

I am sorry to say that I think there is far too much disposition in the profession to sacrifice these teeth recklessly. If they are reasonably good and there is room for them, they ought to be preserved. It is a great misfortune to lose them. They have very much to do with the nutrition of the child—more between six and nine years of age than any other organs in the mouth. They are needed for purposes of mastication. Then they have more to do in prepreserving the beauty of the features than any other teeth in the mouth, unless it be the cuspids—I mean in preserving the features, as they will show when the mouth is closed. As a general rule, where one of them has to be sacrificed for lack of room, it is better to remove all. If all four have to be removed, it need not at all be done at one sitting. I find it is usually too much of a shock for the child. My usual practice is, if I have to begin the sacrifice, to explain it to the parents and to the child too, and you will generally find the child will consent as readily as the parents will. Children at that age, little girls especially, seem to instinctively understand that they are intended to be good-looking, and that their features need to be preserved. Now, these are very scattered remarks, and I know that many members of the Society will say, "Pshaw! I can make a better speech than that myself," and what I got up for was to induce them to do it.

Dr. J. Taft: Mr. President, Dr. Watt has made some very good suggestions in regard to the care of the first permanent molars. Perhaps this question might have been put in another form and be a little more appropriate, as, "What shall be done for the First Permanent Molar Teeth?" However, that is not a special matter. The remarks of Dr. Watt are mainly upon the question in that form, "What shall be done in behalf of the First Permanent Molars?" And here, in reference to the nomenclature, "first permanent molar" is the better name to apply to these teeth. The term "six year molars" is an awkward expression, and one that should be discarded. It is unfortunate to use a number of terms or words meaning the same thing. Let us select the best term, and then use it and discard those that are not so good.

Now, what shall be done for the welfare of the first permanent molars? We must begin where Dr. Watt began his speech—at the early, or formation stage of the tooth, or begin with the mother. Perhaps the treatment of the mother should have reference to this, and to the welfare of all the teeth, prior to the birth of the child. But, then, as dentists ordinarily find them in the mouth, they are all erupted, and what shall be done with them then? During the life of the child, before the appearance of these teeth, reference can be had to the welfare of the teeth, these as well as the rest, in the treatment that shall be given to the child, in its protection from disease-prod n g influences that are so common, and to which all persons are more or less subject. The hygienic regulations under which the child should be brought, should be modified very much, indeed. In many respects children are subject to disease-producing influences in a very marked degree. This is clearly shown by the large amount of infant mortality Why is it that the young of other animals do not have this proportion of death? Why is it that the rule is, in the great majority of cases, that the young of other animals, which we would suppose are not cared for as those of the human kind ought to be, exhibit so great a disparity in the matter of mortality? Forty per cent., perhaps, of the human family die prior to the age of five years, showing that there is something radically wrong in the care, in the exercises of hygienic rules and regulations for their welfare—giving the best nutrition, giving them the best support, surrounding them with the best circumstances, warding off the influences that produce disease; there is something radically wrong in all these particulars. During the formation and development of the teeth children are subjected to diseases of various kinds. Consequently the teeth will certainly undergo deterioration; they will not be developed as perfectly as they should be. Take, for example, the enamel. The organ may be atrophied for want of proper nutrition, and then it will be defective in one or more particulars, and in some instances the organ will be starved, and contraction will take place. Therefore, there is no union at the edges of the enamel in the fissures, and there will be those open points or fissures that we frequently find in the molar teeth. This occurs more frequently on first molars; these fissures are more marked ordinarily in the first molars and in the bicuspids than in any other teeth in the mouth, showing that there was interference at the time of formation of the organs that did not afterward occur. There will be exceptions, of course.

Then, again, defects in the enamel may be manifested in another direction. By want of proper nutrition the enamel may not receive its due amount of material for solidifying or calcification, and that will manifest itself in various ways—either in the pits and grooves, or the imperfect territories in the enamel, which will be shown by the whitened condition, by the change in the appearance of the enamel, defective spots. Sometimes the enamel is defective throughout. Now, the same thing may occur, without any appreciable reason, in the formation of the dentine. Reference should be had to the perfection of the teeth in the nutrition of the child during the time of the growth and development. Though it may be good in all the respects to which I have referred, subsequent privation of nutrition will prevent the due solidification of the teeth. The tooth, at the time of eruption, has nothing like as much calcareous material in it, as it ultimately receives, or will receive under proper nutrition. This, then, is a matter to which attention should be given through the growing period of the child; not only while the tooth-germ is forming, but while the tooth is being developed and growing, and afterward as well.

The period at which these teeth come is a very unfavorable one for the best interests and the welfare of the teeth. They come at a period when the child is susceptible to a great many disturbances, and many perturbations of health occur during this period. It is a time of life that is subject to irritability, and special susceptibility to morbific influences that surround and attach to it; and it is a vulnerable time in which, at any time, disease is liable to fasten upon this organ. Then, these teeth are erupted at a period when the mouth is oftentimes in a state of marked irritation. It is at the time of the throwing off of the temporary teeth, and ordinarily under the best circumstances, there will be somewhat of irritation in the mouth during this period; and in so far as this condition exists are the permanent molars likely to suffer for want of exercise—for want of due use in mastication. The contiguous teeth are loosened, and there is irritation of the gum, and a vigorous use of them would occasion pain, and the inclination of the child, at this time, is to protect the teeth from irritation, and to avoid the use of them in mastication; and the result is that about these molars, accumulations are generally found, and especially unless attention is given to the matter by the parent, or by some one having the child in charge. There will be more or less accumulations of vitiated matter about the teeth. That will be the rule with the first permanent molars, for a year or two after their eruption, a circumstance that directly favors their decay. The child should be stimulated or induced to use these teeth in mastication, from the time they make their appearance, sufficient to prevent the accumulation of foreign substances upon them. When we consider the amount of vitiated material and debris of one kind and another—agglutined mucous, decomposing material, and epithelial scales that cover them during the year or two following their eruption, the only wonder is that they escape as well as they do.

There is one fortunate circumstance in connection with this condition in the mouth, that during the time of this irritation there will be a large inflow of saliva, and a dilution of the deleterious agents. But this is not sufficient, in most cases, to neutralize or overcome the vitiated product and materials in the mouth that operate upon these teeth.

Then, again, the temporary molars are very frequently decayed upon their posterior proximate surfaces, in contact with the anterior proximate surfaces of the permanent molars, holding in contact with the latter, material undergoing decomposition, out of which the materials that will produce decay are formed. They operate, then, upon the anterior surface of the permanent molars. This is a very common point of decay. And in order to avoid or prevent that, special attention should be given to the teeth in this respect. And when there is decay upon the posterior surface of the temporary molar, it should be cut away. The diamond disk is a very good instrument for this purpose, as, with it, the separation can be very readily and easily made without annoyance to the child; whereas, the use of the file would be objectionable, and with many children it would be almost impossible so make such separation with the file. Very scrupulous search should almost be made for decay in the anterior side of the permanent molar, and if there is incip ient decay, let it be at once removed, and let the very best be done in the eradication of decayed points, and a com plete, thorough polishing of the surface, and in the future let them be kept as clean as possible. If there is a point on the crown of these teeth where decay is beginning, that should be filled at once. Taking any ordinary case, by such care the first permanent molars may be, as it seems to me, preserved. But, unfortunately, a large portion of these teeth are allowed to be destroyed before particular attention is given to them.

And, then, from the time of their eruption, they should be well and thoroughly used, which will accomplish two or three things: The friction keeps the teeth free from the deposit to which I referred, and the pressure exercised upon them gives strength to the periosteal attachment, and stimulates the circulation about the roots and into the pulp, and, of course, the bringing into the part an abundant supply of the nutrient material which it demands. In this way the tooth will be better conserved than if it was not properly exercised. This exercise can be given to the teeth in the use of such food as will require sufficient pressure upon the teeth, and sufficient exercise.

Sometimes, when one of these teeth is taken away, the second and third molars will come in and fill up the space, and there will be no loss; but in the great majority of cases this does not occur, to the complete filling up of the space, that has been left by the removal of the first molar, At the age of seventeen, I had two first molars removed, and the spaces stand there to-day as large as when the teeth were taken away; and the teeth did not move forward, or the other teeth go backward to fill up the space, and it has been a very great annoyance from that time to this. But sometimes the second molar will move forward, as a whole, and occupy the space of the first, to a considerable extent, and occasionally almost entirely, but these are exceptional cases. Often the second permanent molar will tip forward, instead of moving forward in an upright position, and then what is the result? You have the posterior corner of the tooth thrown up for occlusion with the opposing tooth above, and a large portion of the surface of the tooth is not used for breaking up the food, and there is imperfect mastication and insalivation of the food. Sometimes the bicuspid tips backward a little, and the same disturbance arises —the right occlusion for thorough mastication is destroyed. In the proper arrangement of these teeth for mastication they come together like the faces of millstones, and they operate, face to face, one upon the other. They are not thrown together, here a corner and there a corner, touching only at a few points.

It is said that the other teeth are preserved by the early removal of these teeth. Well, that depends very much upon circumstances. If proper care is given to the mouth, the other teeth will not suffer because these are retained. It is not necessary for us to cut off one of our fingers in order to save the ethers. No more is it necessary, recklessly, to take away one tooth for the welfare of the other. I know that there may be cases of irregularity when it is necessary to do this. I am speaking now of those cases where the development and arrangement of the teeth are entirely normal. There is no occasion for the removal of the first permanent molars to preserve the other teeth, if the proper care is exercised on their behalf. I regard it as a very great misfortune, indeed, to sacrifice first permanent molars, and I think every effort should be made for their preservation and their retention in the mouth during life.

Dr. G. W. Keely: I would like to ask, if the patient was eight or ten years old, and the four first molars were badly decayed and the pulps exposed, what would you do with them.

Dr. J. Taft. Well, I don't know what I would do exactly; yet I would treat them and endeavor to save them just as earnestly as I would if the person were twenty-five years old. If the person was well developed, and in good condition otherwise, I should certainly endeavor to save the teeth. If I inferred from the general make-up that there would be a breach that would never be repaired in any way,

I should regard it as a very important matter to preserve those teeth as long as possible. The observing dentist will form an opinion when he sees the patient. If it is one of those strong, fixed, unyielding, firm organizations, you will not expect the teeth to come in and fill up the space. In a case of that character I should preserve the teeth as much as I would at any other period of life, just as long as I could keep them free from disease and discomfort to the patient. By the way, I think the material with which those teeth are filled should be carefully selected. I do not think that in every case it is best to fill those teeth at once with gold. Say, from six to seven or eight years of age I should prefer tinfoil ; and perhaps, if the teeth were very sensitive, I might use some more temporary material than that. The teeth generally decay more readily at from seven to eight years of age. I have many times filled teeth with the expectation that the operation would only be temporary, and have found, in many such instances, that the teeth so filled have remained for years and years. There are teeth in this room, now, that I filled twenty-five or twenty-eight years ago, with tin-foil, thinking that they would not last a great while ; and in years afterwards they were found to be no further decayed, and the tin fillings were removed, the teeth filled with gold and the teeth are to-day far better than when they were filled with tin in the first place.

---

